# Supplementary material for: Retrospective identification of key activities in Uganda’s preparedness measures related to the 2018–2020 EVD outbreak in eastern DRC utilizing a framework evaluation tool
Source: PLOS Glob Public Health. 2022 May 11;2(5):e0000428. doi: 10.1371/journal.pgph.0000428 (PMC10021806; doi:10.1371/journal.pgph.0000428)
Supplement: S1 Text — Please, see the list below for the full list of grey literature (143 documents in total) included to inform the framework evaluation tool in no particular order. All grey literature can be found publicly available online or can be made available upon reasonable request to the corresponding author. (DOCX) [file pgph.0000428.s001.docx]

S1 Appendix: Grey Literature References

1. United Nations. Regional Ebola Preparedness: Overview of Needs and Requirements July - December 2019. United Nations; 2019. <https://www.who.int/csr/resources/publications/ebola/regional-preparedness-overview-june-dec-2019.pdf?ua=1>. Accessed 2019.
2. World Health Organization Uganda Country Office, Uganda Ministry of Health. In: WHO Uganda Ebola Virus Preparedness Updates. Ebola Virus Disease Preparedness Update in Uganda as of 3^rd^ August 2018 (12:00 HRS). 2018. <https://www.afro.who.int/sites/default/files/2018-11/WCO%20EVD%20Sitrep%20No%201_0.pdf>. Accessed 17 June 2019.
3. World Health Organization Uganda Country Office, Uganda Ministry of Health. In: WHO Uganda Ebola Virus Preparedness Updates. Highlights of the Ebola Virus Disease Preparedness in Uganda as of 4^th^ August 2018 (12:00 HRS). 2018. <https://www.afro.who.int/sites/default/files/2018-11/WCO%20EVD%20Update%20No2_0.pdf>. Accessed 19 June 2019.
4. World Health Organization Uganda Country Office, Uganda Ministry of Health. In: WHO Uganda Ebola Virus Preparedness Updates. Highlights of the Ebola Virus Disease Preparedness in Uganda as of 6^th^ August 2018 (12:00 HRS). 2018. <https://www.afro.who.int/sites/default/files/2018-11/WCO%20EVD%20Update%20No3_0.pdf>. Accessed 19 June 2019.
5. World Health Organization Uganda Country Office, Uganda Ministry of Health. In: WHO Uganda Ebola Virus Preparedness Updates. Highlights of the Ebola Virus Disease Preparedness in Uganda 7^th^ August 2018 (12:00 HRS). 2018. <https://www.afro.who.int/sites/default/files/2018-11/WCO%20EVD%20Update%20No4_0.pdf>. Accessed 19 June 2019.
6. World Health Organization Uganda Country Office, Uganda Ministry of Health. In: WHO Uganda Ebola Virus Preparedness Updates. Highlights of the Ebola Virus Disease Preparedness in Uganda 8^th^ August 2018 (12:00 HRS) – Update No 5. 2018. <https://www.afro.who.int/sites/default/files/2018-11/WCO%20EVD%20Update%20No5_0.pdf>. Accessed 20 June 2019.
7. World Health Organization Uganda Country Office, Uganda Ministry of Health. In: WHO Uganda Ebola Virus Preparedness Updates. Highlights of the Ebola Virus Disease Preparedness in Uganda 9^th^ August 2018 (12:00 HRS) – Update No 6. 2018. <https://www.afro.who.int/sites/default/files/2018-11/WCO%20EVD%20Update%20No6_0.pdf>. Accessed 21 June 2019.
8. World Health Organization Uganda Country Office, Uganda Ministry of Health. In: WHO Uganda Ebola Virus Preparedness Updates. Highlights of the Ebola Virus Disease Preparedness in Uganda 10^th^ August 2018 (12:00 HRS) – Update No 7. 2018. <https://www.afro.who.int/sites/default/files/2018-11/WCO%20EVD%20Update%20No7_0.pdf>. Accessed 21 June 2019.
9. World Health Organization Uganda Country Office, Uganda Ministry of Health. In: WHO Uganda Ebola Virus Preparedness Updates. Highlights of the Ebola Virus Disease Preparedness in Uganda 11^th^ August 2018 (12:00 HRS) – Update No 8. 2018. <https://www.afro.who.int/sites/default/files/2018-11/WCO%20EVD%20Update%20No8_0.pdf>. Accessed 12 July 2019.
10. World Health Organization Uganda Country Office, Uganda Ministry of Health. In: WHO Uganda Ebola Virus Preparedness Updates. Highlights of the Ebola Virus Disease Preparedness in Uganda 13^th^ August 2018 (12:00 HRS) – Update No 9. 2018. <https://www.afro.who.int/sites/default/files/2018-11/WCO%20EVD%20Update%20No9_0.pdf>. Accessed 12 July 2019.
11. World Health Organization Uganda Country Office, Uganda Ministry of Health. In: WHO Uganda Ebola Virus Preparedness Updates. Highlights of the Ebola Virus Disease Preparedness in Uganda 14^th^ August 2018 (12:00 HRS) – Update No 10. 2018. <https://www.afro.who.int/sites/default/files/2018-11/WCO%20EVD%20Update%20No10_0.pdf>. Accessed 12 July 2019.
12. World Health Organization Uganda Country Office, Uganda Ministry of Health. In: WHO Uganda Ebola Virus Preparedness Updates. Highlights of the Ebola Virus Disease Preparedness in Uganda 15^th^ August 2018 (12:00 HRS) – Update No 11. 2018. <https://www.afro.who.int/sites/default/files/2018-11/WCO%20EVD%20Update%20No11_0.pdf>. Accessed 17 July 2019.
13. World Health Organization Uganda Country Office, Uganda Ministry of Health. In: WHO Uganda Ebola Virus Preparedness Updates. Highlights of the Ebola Virus Disease Preparedness in Uganda 16^th^ August 2018 (12:00 HRS) – Update No 12. 2018. <https://www.afro.who.int/sites/default/files/2018-11/WCO%20EVD%20Update%20No12.pdf>. Accessed 19 July 2019.
14. World Health Organization Uganda Country Office, Uganda Ministry of Health. In: WHO Uganda Ebola Virus Preparedness Updates. Highlights of the Ebola Virus Disease Preparedness in Uganda 17^th^ August 2018 (12:00 HRS) – Update No 13. 2018. <https://www.afro.who.int/sites/default/files/2018-11/WCO%20EVD%20Update%20No13.pdf>. Accessed 16 August 2019.
15. World Health Organization Uganda Country Office, Uganda Ministry of Health. In: WHO Uganda Ebola Virus Preparedness Updates. Highlights of the Ebola Virus Disease Preparedness in Uganda 18^th^ August 2018 (12:00 HRS) – Update No 14. 2018. <https://www.afro.who.int/sites/default/files/2018-11/EVD%20Preparedness%20Update%20No%2014_0.pdf>. Accessed 16 August 2019.
16. World Health Organization Uganda Country Office, Uganda Ministry of Health. In: WHO Uganda Ebola Virus Preparedness Updates. Highlights of the Ebola Virus Disease Preparedness in Uganda 19^th^ August 2018 (12:00 HRS) – Update No 15. 2018. <https://www.afro.who.int/sites/default/files/2018-11/EVD%20Update%20No%2015_0.pdf>. Accessed 16 August 2019.
17. World Health Organization Uganda Country Office, Uganda Ministry of Health. In: WHO Uganda Ebola Virus Preparedness Updates. Highlights of the Ebola Virus Disease Preparedness in Uganda 22^nd^ August 2018 (12:00 HRS) – Update No 16. 2018. <https://www.afro.who.int/sites/default/files/2018-11/WCO%20EVD%20Update%20No%2016_0.pdf>. Accessed 20 August 2019.
18. World Health Organization Uganda Country Office, Uganda Ministry of Health. In: WHO Uganda Ebola Virus Preparedness Updates. Highlights of the Ebola Virus Disease Preparedness in Uganda 24^th^ August 2018 (12:00 HRS) – Update No 17. 2018. <https://www.afro.who.int/sites/default/files/2018-11/EVD%20Preparedness%20Update%20No%2017_0.pdf>. Accessed 21 August 2019.
19. World Health Organization Uganda Country Office, Uganda Ministry of Health. In: WHO Uganda Ebola Virus Preparedness Updates. Highlights of the Ebola Virus Disease Preparedness in Uganda 29^th^ August 2018 (12:00 HRS) – Update No 19. 2018. <https://www.afro.who.int/sites/default/files/2018-11/EVD%20Preparedness%20Update%20No%2019_0.pdf>. Accessed 23 August 2019.
20. World Health Organization Uganda Country Office, Uganda Ministry of Health. In: WHO Uganda Ebola Virus Preparedness Updates. Highlights of the Ebola Virus Disease Preparedness in Uganda 31^st^ August 2018 (12:00 HRS) – Update No 21. 2018. <https://www.afro.who.int/sites/default/files/2018-11/EVD%20Preparedness%20Update%20No%2021_0.pdf>. Accessed 23 August 2019.
21. World Health Organization Uganda Country Office, Uganda Ministry of Health. In: WHO Uganda Ebola Virus Preparedness Updates. Highlights of the Ebola Virus Disease Preparedness in Uganda 11^th^ September 2018 (12:00 HRS) – Update No 23. 2018. <https://www.afro.who.int/sites/default/files/2018-11/EVD%20Update%20No%2023_0.pdf>. Accessed 6 June 2019.
22. World Health Organization Uganda Country Office, Uganda Ministry of Health. In: WHO Uganda Ebola Virus Preparedness Updates. Highlights of the Ebola Virus Disease Preparedness in Uganda 18^th^ September 2018 (12:00 HRS) – Update No 25. 2018. <https://www.afro.who.int/sites/default/files/2018-11/EVD%20Preparedness%20Update%20No%2025_0.pdf>. Accessed 6 June 2019.
23. World Health Organization Uganda Country Office, Uganda Ministry of Health. In: WHO Uganda Ebola Virus Preparedness Updates. Highlights of the Ebola Virus Disease Preparedness in Uganda 21^st^ September 2018 (12:00 HRS) – Update No 26. 2018. <https://www.afro.who.int/sites/default/files/2018-11/EVD%20Update%20No%2026_0.pdf>. Accessed 6 June 2019.
24. World Health Organization Uganda Country Office, Uganda Ministry of Health. In: WHO Uganda Ebola Virus Preparedness Updates. Highlights of the Ebola Virus Disease Preparedness in Uganda 25^th^ September 2018 (12:00 HRS) – Update No 27. 2018. <https://www.afro.who.int/sites/default/files/2018-11/EVD%20Update%20No%2027_0.pdf>. Accessed 6 June 2019.
25. World Health Organization Uganda Country Office, Uganda Ministry of Health. In: WHO Uganda Ebola Virus Preparedness Updates. Highlights of the Ebola Virus Disease Preparedness in Uganda 28^th^ September 2018 (12:00 HRS) – Update No 28. 2018. <https://www.afro.who.int/sites/default/files/2018-11/EVD%20Update%20No%2028_0.pdf>. Accessed 6 June 2019.
26. World Health Organization Uganda Country Office, Uganda Ministry of Health. In: WHO Uganda Ebola Virus Preparedness Updates. Highlights of the Ebola Virus Disease Preparedness in Uganda 2^nd^ October 2018 (12:00 HRS) – Update No 29. 2018. <https://www.afro.who.int/sites/default/files/2018-11/EVD%20Update%20No%2029_0.pdf>. Accessed 6 June 2019.
27. World Health Organization Uganda Country Office, Uganda Ministry of Health. In: WHO Uganda Ebola Virus Preparedness Updates. Highlights of the Ebola Virus Disease Preparedness in Uganda 5^th^ October 2018 (12:00 HRS) – Update No 30. 2018. <https://www.afro.who.int/sites/default/files/2018-11/EVD%20Update%20No%2030_0.pdf>. Accessed 6 June 2019.
28. World Health Organization Uganda Country Office, Uganda Ministry of Health. In: WHO Uganda Ebola Virus Preparedness Updates. Highlights of the Ebola Virus Disease Preparedness in Uganda 8^th^ October 2018 (12:00 HRS) – Update No 31. 2018. <https://www.afro.who.int/sites/default/files/2018-11/WCO%20EVD%20Update%20No%2031_0.pdf>. Accessed 6 June 2019.
29. World Health Organization Uganda Country Office, Uganda Ministry of Health. In: WHO Uganda Ebola Virus Preparedness Updates. Highlights of the Ebola Virus Disease Preparedness in Uganda 10^th^ October 2018 (12:00 HRS) – Update No 32. 2018. <https://www.afro.who.int/sites/default/files/2018-11/EVD%20Update%20No%2032_0.pdf>. Accessed 6 June 2019.
30. World Health Organization Uganda Country Office, Uganda Ministry of Health. In: WHO Uganda Ebola Virus Preparedness Updates. Highlights of the Ebola Virus Disease Preparedness in Uganda 11^th^ October 2018 (12:00 HRS) – Update No 33. 2018. <https://www.afro.who.int/sites/default/files/2018-11/EVD%20Update%20No%2033_0.pdf>. Accessed 6 June 2019.
31. World Health Organization Uganda Country Office, Uganda Ministry of Health. In: WHO Uganda Ebola Virus Preparedness Updates. Highlights of the Ebola Virus Disease Preparedness in Uganda 12^th^ October 2018 (12:00 HRS) – Update No 34. 2018. <https://www.afro.who.int/sites/default/files/2018-11/EVD%20Update%20No%2034_0.pdf>. Accessed 6 June 2019.
32. World Health Organization Uganda Country Office, Uganda Ministry of Health. In: WHO Uganda Ebola Virus Preparedness Updates. Highlights of the Ebola Virus Disease Preparedness in Uganda 15^th^ October 2018 (12:00 HRS) – Update No 35. 2018. <https://www.afro.who.int/sites/default/files/2018-11/EVD%20Update%20No%2035_0.pdf>. Accessed 7 June 2019.
33. World Health Organization Uganda Country Office, Uganda Ministry of Health. In: WHO Uganda Ebola Virus Preparedness Updates. Highlights of the Ebola Virus Disease Preparedness in Uganda 16^th^ October 2018 (12:00 HRS) – Update No 36. 2018. <https://www.afro.who.int/sites/default/files/2018-11/WCO%20EVD%20Update%20No%2036_0.pdf>. Accessed 7 June 2019.
34. World Health Organization Uganda Country Office, Uganda Ministry of Health. In: WHO Uganda Ebola Virus Preparedness Updates. Highlights of the Ebola Virus Disease Preparedness in Uganda 19^th^ October 2018 (12:00 HRS) – Update No 37. 2018. <https://www.afro.who.int/sites/default/files/2018-11/WCO%20EVD%20Update%20No%2037_0.pdf>. Accessed 7 June 2019.
35. World Health Organization Uganda Country Office, Uganda Ministry of Health. In: WHO Uganda Ebola Virus Preparedness Updates. Highlights of the Ebola Virus Disease Preparedness in Uganda 22^nd^ October 2018 (12:00 HRS) – Update No 38. 2018. <https://www.afro.who.int/sites/default/files/2018-11/WCO%20EVD%20Update%20No%2038.pdf>. Accessed 7 June 2019.
36. World Health Organization Uganda Country Office, Uganda Ministry of Health. In: WHO Uganda Ebola Virus Preparedness Updates. Highlights of the Ebola Virus Disease Preparedness in Uganda 23^rd^ October 2018 (12:00 HRS) – Update No 39. 2018. <https://www.afro.who.int/sites/default/files/2018-11/EVD%20Update%20No%2039_0.pdf>. Accessed 7 June 2019.
37. World Health Organization Uganda Country Office, Uganda Ministry of Health. In: WHO Uganda Ebola Virus Preparedness Updates. Highlights of the Ebola Virus Disease Preparedness in Uganda 25^th^ October 2018 (12:00 HRS) – Update No 40. 2018. <https://www.afro.who.int/sites/default/files/2018-11/UGA%20EVD%20Update%20No%2040_0.pdf>. Accessed 7 June 2019.
38. World Health Organization Uganda Country Office, Uganda Ministry of Health. In: WHO Uganda Ebola Virus Preparedness Updates. Highlights of the Ebola Virus Disease Preparedness in Uganda 26^th^ October 2018 (12:00 HRS) – Update No 41. 2018. <https://www.afro.who.int/sites/default/files/2018-11/UGA%20EVD%20Update%20No%2041_0.pdf>. Accessed 7 June 2019.
39. World Health Organization Uganda Country Office, Uganda Ministry of Health. In: WHO Uganda Ebola Virus Preparedness Updates. Highlights of the Ebola Virus Disease Preparedness in Uganda 29^th^ October 2018 (12:00 HRS) – Update No 42. 2018. <https://www.afro.who.int/sites/default/files/2018-11/UGA%20EVD%20Update%20No%2042.pdf>. Accessed 7 June 2019.
40. World Health Organization Uganda Country Office, Uganda Ministry of Health. In: WHO Uganda Ebola Virus Preparedness Updates. Highlights of the Ebola Virus Disease Preparedness in Uganda 30^th^ October 2018 (12:00 HRS) – Update No 43. 2018. <https://www.afro.who.int/sites/default/files/2018-11/UGA%20EVD%20Update%20No%2043_0.pdf>. Accessed 7 June 2019.
41. World Health Organization Uganda Country Office, Uganda Ministry of Health. In: WHO Uganda Ebola Virus Preparedness Updates. Highlights of the Ebola Virus Disease Preparedness in Uganda 31^st^ October 2018 (12:00 HRS) – Update No 44. 2018. <https://www.afro.who.int/sites/default/files/2018-11/EVD%20Update%20No%2044_0.pdf>. Accessed 7 June 2019.
42. World Health Organization Uganda Country Office, Uganda Ministry of Health. In: WHO Uganda Ebola Virus Preparedness Updates. Highlights of the Ebola Virus Disease Preparedness in Uganda 1^st^ November 2018 (12:00 HRS) – Update No 45. 2018. <https://www.afro.who.int/sites/default/files/2018-11/EVD%20Update%20No%2045_0.pdf>. Accessed 7 June 2019.
43. World Health Organization Uganda Country Office, Uganda Ministry of Health. In: WHO Uganda Ebola Virus Preparedness Updates. Highlights of the Ebola Virus Disease Preparedness in Uganda 2^nd^ November 2018 (12:00 HRS) – Update No 46. 2018. <https://www.afro.who.int/sites/default/files/2018-11/EVD%20Update%20No%2046_0.pdf>. Accessed 7 June 2019.
44. World Health Organization Uganda Country Office, Uganda Ministry of Health. In: WHO Uganda Ebola Virus Preparedness Updates. Highlights of the Ebola Virus Disease Preparedness in Uganda 5^th^ November 2018 (12:00 HRS) – Update No 47. 2018. <https://www.afro.who.int/sites/default/files/2018-11/EVD%20Update%20No%2047_0.pdf>. Accessed 7 June 2019.
45. World Health Organization Uganda Country Office, Uganda Ministry of Health. In: WHO Uganda Ebola Virus Preparedness Updates. Highlights of the Ebola Virus Disease Preparedness in Uganda 6^th^ November 2018 (12:00 HRS) – Update No 48. 2018. <https://www.afro.who.int/sites/default/files/2018-11/EVD%20Update%20No%2048_0.pdf>. Accessed 7 June 2019.
46. World Health Organization Uganda Country Office, Uganda Ministry of Health. In: WHO Uganda Ebola Virus Preparedness Updates. Highlights of the Ebola Virus Disease Preparedness in Uganda 7^th^ November 2018 (12:00 HRS) – Update No 49. 2018. <https://www.afro.who.int/sites/default/files/2018-11/EVD%20District%20level%20Update%20No%2049_0.pdf>. Accessed 7 June 2019.
47. World Health Organization Uganda Country Office, Uganda Ministry of Health. In: WHO Uganda Ebola Virus Preparedness Updates. Highlights of the Ebola Virus Disease Preparedness in Uganda 8^th^ November 2018 (12:00 HRS) – Update No 50. 2018. <https://www.afro.who.int/sites/default/files/2018-11/UGA%20EVD%20Update%20No%2050_0.pdf>. Accessed 7 June 2019.
48. World Health Organization Uganda Country Office, Uganda Ministry of Health. In: WHO Uganda Ebola Virus Preparedness Updates. Highlights of the Ebola Virus Disease Preparedness in Uganda 9^th^ November 2018 (12:00 HRS) – Update No 51. 2018. <https://www.afro.who.int/sites/default/files/2018-11/EVD%20Field%20and%20national%20Update%20No%2051_0.pdf>. Accessed 7 June 2019.
49. World Health Organization Uganda Country Office, Uganda Ministry of Health. In: WHO Uganda Ebola Virus Preparedness Updates. Highlights of the Ebola Virus Disease Preparedness in Uganda 12^th^ November 2018 (12:00 HRS) – Update No 52. 2018. <https://www.afro.who.int/sites/default/files/2018-11/UGA%20EVD%20Update%20No%2052_0.pdf>. Accessed 7 June 2019.
50. World Health Organization Uganda Country Office, Uganda Ministry of Health. In: WHO Uganda Ebola Virus Preparedness Updates. Highlights of the Ebola Virus Disease Preparedness in Uganda 14^th^ November 2018 (12:00 HRS) – Update No 54. 2018. <https://www.afro.who.int/sites/default/files/2018-11/EVD%20Update%20No%2054_0.pdf>. Accessed 7 June 2019.
51. World Health Organization Uganda Country Office, Uganda Ministry of Health. In: WHO Uganda Ebola Virus Preparedness Updates. Highlights of the Ebola Virus Disease Preparedness in Uganda 16^th^ November 2018 (12:00 HRS) – Update No 56. 2018. <https://www.afro.who.int/sites/default/files/2018-11/UGA%20EVD%20Update%20No%2056.pdf>. Accessed 7 June 2019.
52. World Health Organization Uganda Country Office, Uganda Ministry of Health. In: WHO Uganda Ebola Virus Preparedness Updates. Highlights of the Ebola Virus Disease Preparedness in Uganda 19^th^ November 2018 (12:00 HRS) – Update No 57. 2018. <https://www.afro.who.int/sites/default/files/2018-11/EVD%20Update%20No%2057.pdf>. Accessed 7 June 2019.
53. World Health Organization Uganda Country Office, Uganda Ministry of Health. In: WHO Uganda Ebola Virus Preparedness Updates. Highlights of the Ebola Virus Disease Preparedness in Uganda 20^th^ November 2018 (12:00 HRS) – Update No 58. 2018. <https://www.afro.who.int/sites/default/files/2018-11/EVD%20Update%20No%2058.pdf>. Accessed 7 June 2019.
54. World Health Organization Uganda Country Office, Uganda Ministry of Health. In: WHO Uganda Ebola Virus Preparedness Updates. Highlights of the Ebola Virus Disease Preparedness in Uganda 23^rd^ November 2018 (12:00 HRS) – Update No 60. 2018. <https://www.afro.who.int/sites/default/files/2018-12/UGA%20EVD%20Update%20No%2060.pdf>. Accessed 7 June 2019.
55. World Health Organization Uganda Country Office, Uganda Ministry of Health. In: WHO Uganda Ebola Virus Preparedness Updates. Highlights of the Ebola Virus Disease Preparedness in Uganda 28^th^ November 2018 (12:00 HRS) – Update No 62. 2018. <https://www.afro.who.int/sites/default/files/2018-12/EVD%20Update%20No%2062.pdf>. Accessed 7 June 2019.
56. World Health Organization Uganda Country Office, Uganda Ministry of Health. In: WHO Uganda Ebola Virus Preparedness Updates. Highlights of the Ebola Virus Disease Preparedness in Uganda 29^th^ November 2018 (12:00 HRS) – Update No 63. 2018. <https://www.afro.who.int/sites/default/files/2018-12/EVD%20Update%20No%2063.pdf>. Accessed 7 June 2019.
57. World Health Organization Uganda Country Office, Uganda Ministry of Health. In: WHO Uganda Ebola Virus Preparedness Updates. Highlights of the Ebola Virus Disease Preparedness in Uganda 30^th^ November 2018 (12:00 HRS) – Update No 64. 2018. <https://www.afro.who.int/sites/default/files/2018-12/EVD%20Update%20No%2064.pdf>. Accessed 7 June 2019.
58. World Health Organization Uganda Country Office, Uganda Ministry of Health. In: WHO Uganda Ebola Virus Preparedness Updates. Highlights of the Ebola Virus Disease Preparedness in Uganda 29^th^ January 2019 (12:00 HRS) – Update No 78. 2018. <https://www.afro.who.int/sites/default/files/2019-02/UGA%20EVD%20Update%20No%2078.pdf>. Accessed 7 June 2019.
59. World Health Organization Uganda Country Office, Uganda Ministry of Health. In: WHO Uganda Ebola Virus Preparedness Updates. Highlights of the Ebola Virus Disease Preparedness in Uganda 30^th^ January 2019 (12:00 HRS) – Update No 79. 2018. <https://www.afro.who.int/sites/default/files/2019-02/EVD%20Update%20No%2079.pdf>. Accessed 7 June 2019.
60. World Health Organization Uganda Country Office, Uganda Ministry of Health. In: WHO Uganda Ebola Virus Preparedness Updates. Highlights of the Ebola Virus Disease Preparedness in Uganda 5^th^ February 2019 (12:00 HRS) – Update No 82. 2018. <https://www.afro.who.int/sites/default/files/2019-02/EVD%20Update%20No%2082.pdf>. Accessed 7 June 2019.
61. World Health Organization Uganda Country Office, Uganda Ministry of Health. In: WHO Uganda Ebola Virus Preparedness Updates. Highlights of the Ebola Virus Disease Preparedness in Uganda 5^th^ February 2019 (12:00 HRS) – Update No 83. 2018. <https://www.afro.who.int/sites/default/files/2019-02/EVD%20Update%20No%2083.pdf>. Accessed 7 June 2019.
62. World Health Organization Uganda Country Office, Uganda Ministry of Health. In: WHO Uganda Ebola Virus Preparedness Updates. Highlights of the Ebola Virus Disease Preparedness in Uganda 6^th^ February 2019 (12:00 HRS) – Update No 84. 2018. <https://www.afro.who.int/sites/default/files/2019-02/EVD%20Update%20No%2084.pdf>. Accessed 7 June 2019.
63. World Health Organization Uganda Country Office, Uganda Ministry of Health. In: WHO Uganda Ebola Virus Preparedness Updates. Highlights of the Ebola Virus Disease Preparedness in Uganda 8^th^ February 2019 (12:00 HRS) – Update No 85. 2018. <https://www.afro.who.int/sites/default/files/2019-02/EVD%20Update%20No%2085.pdf>. Accessed 7 June 2019.
64. World Health Organization Uganda Country Office, Uganda Ministry of Health. In: WHO Uganda Ebola Virus Preparedness Updates. Highlights of the Ebola Virus Disease Preparedness in Uganda 14^th^ February 2019 (12:00 HRS) – Update No 87. 2018. <https://www.afro.who.int/sites/default/files/2019-02/EVD%20Update%20No%2087.pdf>. Accessed 7 June 2019.
65. World Health Organization Uganda Country Office, Uganda Ministry of Health. In: WHO Uganda Ebola Virus Preparedness Updates. Highlights of the Ebola Virus Disease Preparedness in Uganda 18^th^ February 2019 (12:00 HRS) – Update No 88. 2018. <https://www.afro.who.int/sites/default/files/2019-02/EVD%20Update%20No%2088.pdf>. Accessed 7 June 2019.
66. World Health Organization Uganda Country Office, Uganda Ministry of Health. In: WHO Uganda Ebola Virus Preparedness Updates. Highlights of the Ebola Virus Disease Preparedness in Uganda 20^th^ February 2019 (12:00 HRS) – Update No 89. 2018. <https://www.afro.who.int/sites/default/files/2019-02/EVD%20Update%20No%2089.pdf>. Accessed 7 June 2019.
67. World Health Organization Uganda Country Office, Uganda Ministry of Health. In: WHO Uganda Ebola Virus Preparedness Updates. Highlights of the Ebola Virus Disease Preparedness in Uganda 21^st^ February 2019 (12:00 HRS) – Update No 90. 2018. <https://www.afro.who.int/sites/default/files/2019-02/EVD%20Update%20No%2090.pdf>. Accessed 7 June 2019.
68. World Health Organization Uganda Country Office, Uganda Ministry of Health. In: WHO Uganda Ebola Virus Preparedness Updates. Highlights of the Ebola Virus Disease Preparedness in Uganda 22^nd^ February 2019 (12:00 HRS) – Update No 91. 2018. <https://www.afro.who.int/sites/default/files/2019-02/EVD%20Update%20No%2091.pdf>. Accessed 7 June 2019.
69. World Health Organization Uganda Country Office, Uganda Ministry of Health. In: WHO Uganda Ebola Virus Preparedness Updates. Highlights of the Ebola Virus Disease Preparedness in Uganda 27^th^ February 2019 (12:00 HRS) – Update No 92. 2018. <https://www.afro.who.int/sites/default/files/2019-03/EVD%20Update%20No%2092.pdf>. Accessed 7 June 2019.
70. World Health Organization Uganda Country Office, Uganda Ministry of Health. In: WHO Uganda Ebola Virus Preparedness Updates. Highlights of the Ebola Virus Disease Preparedness in Uganda 28^th^ February 2019 (12:00 HRS) – Update No 93. 2018. <https://www.afro.who.int/sites/default/files/2019-03/EVD%20Update%20No%2093.pdf>. Accessed 7 June 2019.
71. World Health Organization Uganda Country Office, Uganda Ministry of Health. In: WHO Uganda Ebola Virus Preparedness Updates. Highlights of the Ebola Virus Disease Preparedness in Uganda 28^th^ February 2019 (12:00 HRS) – Update No 94. 2018. <https://www.afro.who.int/sites/default/files/2019-03/EVD%20Update%20No%2094.pdf>. Accessed 7 June 2019.
72. World Health Organization Uganda Country Office, Uganda Ministry of Health. In: WHO Uganda Ebola Virus Preparedness Updates. Highlights of the Ebola Virus Disease Preparedness in Uganda 5^th^ March 2019 (12:00 HRS) – Update No 95. 2018. <https://www.afro.who.int/sites/default/files/2019-03/EVD%20Update%20No%2095.pdf>. Accessed 7 June 2019.
73. World Health Organization Uganda Country Office, Uganda Ministry of Health. In: WHO Uganda Ebola Virus Preparedness Updates. Highlights of the Ebola Virus Disease Preparedness in Uganda 6^th^ March 2019 (12:00 HRS) – Update No 96. 2018. <https://www.afro.who.int/sites/default/files/2019-03/EVD%20Update%20No%2096.pdf>. Accessed 7 June 2019.
74. World Health Organization Uganda Country Office, Uganda Ministry of Health. In: WHO Uganda Ebola Virus Preparedness Updates. Highlights of the Ebola Virus Disease Preparedness in Uganda 7^th^ March 2019 (12:00 HRS) – Update No 97. 2018. <https://www.afro.who.int/sites/default/files/2019-03/EVD%20Update%20No%2097.pdf>. Accessed 7 June 2019.
75. World Health Organization Uganda Country Office, Uganda Ministry of Health. In: WHO Uganda Ebola Virus Preparedness Updates. Highlights of the Ebola Virus Disease Preparedness in Uganda 11^th^ March 2019 (12:00 HRS) – Update No 98. 2018. <https://www.afro.who.int/sites/default/files/2019-03/EVD%20Update%20No%2098.pdf>. Accessed 7 June 2019.
76. World Health Organization Uganda Country Office, Uganda Ministry of Health. In: WHO Uganda Ebola Virus Preparedness Updates. Highlights of the Ebola Virus Disease Preparedness in Uganda 12^th^ March 2019 (12:00 HRS) – Update No 99. 2018. <https://www.afro.who.int/sites/default/files/2019-03/EVD%20Update%20No%2099.pdf>. Accessed 7 June 2019.
77. World Health Organization Uganda Country Office, Uganda Ministry of Health. In: WHO Uganda Ebola Virus Preparedness Updates. Highlights of the Ebola Virus Disease Preparedness in Uganda 14^th^ March 2019 (12:00 HRS) – Update No 100. 2018. <https://www.afro.who.int/sites/default/files/2019-03/EVD%20Update%20No%20100.pdf>. Accessed 7 June 2019.
78. World Health Organization Uganda Country Office, Uganda Ministry of Health. In: WHO Uganda Ebola Virus Preparedness Updates. Highlights of the Ebola Virus Disease Preparedness in Uganda 14^th^ March 2019 (12:00 HRS) – Update No 101. 2018. <https://www.afro.who.int/sites/default/files/2019-03/EVD%20Update%20No%20101.pdf>. Accessed 7 June 2019.
79. World Health Organization Uganda Country Office, Uganda Ministry of Health. In: WHO Uganda Ebola Virus Preparedness Updates. Highlights of the Ebola Virus Disease Preparedness in Uganda 18^th^ March 2019 (12:00 HRS) – Update No 102. 2018. <https://www.afro.who.int/sites/default/files/2019-03/EVD%20Update%20No%20102_0.pdf>. Accessed 7 June 2019.
80. World Health Organization Uganda Country Office, Uganda Ministry of Health. In: WHO Uganda Ebola Virus Preparedness Updates. Highlights of the Ebola Virus Disease Preparedness in Uganda 19^th^ March 2019 (12:00 HRS) – Update No 103. 2018. <https://www.afro.who.int/sites/default/files/2019-03/EVD%20Update%20No%20103.pdf>. Accessed 7 June 2019.
81. World Health Organization Uganda Country Office, Uganda Ministry of Health. In: WHO Uganda Ebola Virus Preparedness Updates. Highlights of the Ebola Virus Disease Preparedness in Uganda 19^th^ March 2019 (12:00 HRS) – Update No 104. 2018. <https://www.afro.who.int/sites/default/files/2019-03/EVD%20Update%20No%20104.pdf>. Accessed 7 June 2019.
82. World Health Organization Uganda Country Office, Uganda Ministry of Health. In: WHO Uganda Ebola Virus Preparedness Updates. Highlights of the Ebola Virus Disease Preparedness in Uganda 21^st^ March 2019 (12:00 HRS) – Update No 105. 2018. <https://www.afro.who.int/sites/default/files/2019-03/UGA%20EVD%20Update%20No%20105.pdf>. Accessed 7 June 2019.
83. World Health Organization Uganda Country Office, Uganda Ministry of Health. In: WHO Uganda Ebola Virus Preparedness Updates. Highlights of the Ebola Virus Disease Preparedness in Uganda 22^nd^ March 2019 (12:00 HRS) – Update No 106. 2018. <https://www.afro.who.int/sites/default/files/2019-03/EVD%20Update%20No%20106.pdf>. Accessed 7 June 2019.
84. World Health Organization Uganda Country Office, Uganda Ministry of Health. In: WHO Uganda Ebola Virus Preparedness Updates. Highlights of the Ebola Virus Disease Preparedness in Uganda 25^th^ March 2019 (12:00 HRS) – Update No 107. 2018. <https://www.afro.who.int/sites/default/files/2019-03/EVD%20Update%20No%20107.pdf>. Accessed 7 June 2019.
85. World Health Organization Uganda Country Office, Uganda Ministry of Health. In: WHO Uganda Ebola Virus Preparedness Updates. Highlights of the Ebola Virus Disease Preparedness in Uganda 26^th^ March 2019 (12:00 HRS) – Update No 108. 2018. <https://www.afro.who.int/sites/default/files/2019-03/EVD%20Update%20No%20108.pdf>. Accessed 7 June 2019.
86. World Health Organization Uganda Country Office, Uganda Ministry of Health. In: WHO Uganda Ebola Virus Preparedness Updates. Highlights of the Ebola Virus Disease Preparedness in Uganda 27^th^ March 2019 (12:00 HRS) – Update No 109. 2018. <https://www.afro.who.int/sites/default/files/2019-03/EVD%20Update%20No%20109.pdf>. Accessed 7 June 2019.
87. World Health Organization Uganda Country Office, Uganda Ministry of Health. In: WHO Uganda Ebola Virus Preparedness Updates. Highlights of the Ebola Virus Disease Preparedness in Uganda 28^th^ March 2019 (12:00 HRS) – Update No 110. 2018. <https://www.afro.who.int/sites/default/files/2019-04/EVD%20Update%20No%20110.pdf>. Accessed 7 June 2019.
88. World Health Organization Uganda Country Office, Uganda Ministry of Health. In: WHO Uganda Ebola Virus Preparedness Updates. Highlights of the Ebola Virus Disease Preparedness in Uganda 29^th^ March 2019 (12:00 HRS) – Update No 110. 2018. <https://www.afro.who.int/sites/default/files/2019-04/EVD%20Update%20No%20111.pdf>. Accessed 7 June 2019.
89. World Health Organization Uganda Country Office, Uganda Ministry of Health. In: WHO Uganda Ebola Virus Preparedness Updates. Highlights of the Ebola Virus Disease Preparedness in Uganda 29^th^ March 2019 (12:00 HRS) – Update No 112. 2018. <https://www.afro.who.int/sites/default/files/2019-04/EVD%20Update%20No%20112.pdf>. Accessed 7 June 2019.
90. World Health Organization Uganda Country Office, Uganda Ministry of Health. In: WHO Uganda Ebola Virus Preparedness Updates. Highlights of the Ebola Virus Disease Preparedness in Uganda 2^nd^ April 2019 (12:00 HRS) – Update No 113. 2018. <https://www.afro.who.int/sites/default/files/2019-04/EVD%20Update%20No%20113.pdf>. Accessed 7 June 2019.
91. World Health Organization Uganda Country Office, Uganda Ministry of Health. In: WHO Uganda Ebola Virus Preparedness Updates. Highlights of the Ebola Virus Disease Preparedness in Uganda 3^rd^ April 2019 (12:00 HRS) – Update No 114. 2018. <https://www.afro.who.int/sites/default/files/2019-04/EVD%20Update%20No%20114.pdf>. Accessed 7 June 2019.
92. World Health Organization Uganda Country Office, Uganda Ministry of Health. In: WHO Uganda Ebola Virus Preparedness Updates. Highlights of the Ebola Virus Disease Preparedness in Uganda 20^th^ May 2019 (12:00 HRS) – Update No 118. 2018. <https://www.afro.who.int/sites/default/files/2019-05/EVD%20Update%20No%20117.pdf>. Accessed 7 June 2019.
93. World Health Organization Uganda Country Office, Uganda Ministry of Health. In: WHO Uganda Ebola Virus Preparedness Updates. Highlights of the Ebola Virus Disease Preparedness in Uganda 21^st^ May 2019 (12:00 HRS) – Update No 118. 2018. <https://www.afro.who.int/sites/default/files/2019-05/EVD%20Update%20No%20118.pdf>. Accessed 7 June 2019.
94. World Health Organization Uganda Country Office, Uganda Ministry of Health. In: WHO Uganda Ebola Virus Preparedness Updates. Highlights of the Ebola Virus Disease Preparedness in Uganda 22^nd^ May 2019 (12:00 HRS) – Update No 119. 2018. <https://www.afro.who.int/sites/default/files/2019-05/EVD%20Update%20No%20119.pdf>. Accessed 7 June 2019.
95. World Health Organization Uganda Country Office, Uganda Ministry of Health. In: WHO Uganda Ebola Virus Preparedness Updates. Highlights of the Ebola Virus Disease Preparedness in Uganda 23^rd^ May 2019 (12:00 HRS) – Update No 120. 2018. <https://www.afro.who.int/sites/default/files/2019-05/EVD%20Update%20No%20120.pdf>. Accessed 7 June 2019.
96. World Health Organization Uganda Country Office, Uganda Ministry of Health. In: WHO Uganda Ebola Virus Preparedness Updates. Highlights of the Ebola Virus Disease Preparedness in Uganda 28^th^ May 2019 (12:00 HRS) – Update No 121. 2018. <https://www.afro.who.int/sites/default/files/2019-05/EVD%20Update%20No%20121.pdf>. Accessed 7 June 2019.
97. World Health Organization Uganda Country Office, Uganda Ministry of Health. In: WHO Uganda Ebola Virus Preparedness Updates. Highlights of the Ebola Virus Disease Preparedness in Uganda 30^th^ May 2019 (12:00 HRS) – Update No 122. 2018. <https://www.afro.who.int/sites/default/files/2019-05/EVD%20Update%20No%20122.pdf>. Accessed 7 June 2019.
98. World Health Organization Uganda Country Office, Uganda Ministry of Health. In: Ebola Virus Disease Outbreak Uganda Situation Reports. Ebola Virus Disease in Uganda Situation Report 12 June 2019 Sitrep #01. 2019. <https://www.afro.who.int/sites/default/files/2019-07/Ebola%20Virus%20Disease%20Sitrep%201%2012th%20June%202019_0.pdf>. Accessed 2019.
99. World Health Organization Uganda Country Office, Uganda Ministry of Health. In: Ebola Virus Disease Outbreak Uganda Situation Reports. Ebola Virus Disease in Uganda Situation Report 13 June 2019 Sitrep #02. 2019. <https://www.afro.who.int/sites/default/files/2019-07/Ebola%20Virus%20Disease%20Sitrep%202%2013th%20June%202019_0.pdf>. Accessed 2019.
100. World Health Organization Uganda Country Office, Uganda Ministry of Health. In: Ebola Virus Disease Outbreak Uganda Situation Reports. Ebola Virus Disease in Uganda Situation Report 14 June 2019 SitRep #03. 2019. <https://www.afro.who.int/sites/default/files/2019-07/Ebola%20Virus%20Disease%20Sitrep%203%2014th%20June%202019_0.pdf>. Accessed 2019.
101. World Health Organization Uganda Country Office, Uganda Ministry of Health. In: Ebola Virus Disease Outbreak Uganda Situation Reports. Ebola Virus Disease in Uganda Situation Report 16 June 2019 Sitrep #04. 2019. <https://www.afro.who.int/sites/default/files/2019-07/Ebola%20Virus%20Disease%20Sitrep%204%2016th%20June%202019_0.pdf>. Accessed 2019.
102. World Health Organization Uganda Country Office, Uganda Ministry of Health. In: Ebola Virus Disease Outbreak Uganda Situation Reports. Ebola Virus Disease in Uganda Situation Report 16 June 2019 SitRep #05. 2019. <https://www.afro.who.int/sites/default/files/2019-07/Ebola%20Virus%20Disease%20Sitrep%205%2017th%20June%202019_0.pdf>. Accessed 2019.
103. World Health Organization Uganda Country Office, Uganda Ministry of Health. In: Ebola Virus Disease Outbreak Uganda Situation Reports. Ebola Virus Disease in Uganda Situation Report 17 June 2019 as of 20 00 Hrs SitRep #06. 2019. <https://www.afro.who.int/sites/default/files/2019-07/Ebola%20Virus%20Disease%20Sitrep%206%2018th%20June%202019_0.pdf>. Accessed 2019.
104. World Health Organization Uganda Country Office, Uganda Ministry of Health. In: Ebola Virus Disease Outbreak Uganda Situation Reports. Ebola Virus Disease in Uganda Situation Report 18 June 2019 as of 20 00 Hrs SitRep #07. 2019. <https://www.afro.who.int/sites/default/files/2019-07/Ebola%20Virus%20Disease%20Sitrep%207%2019th%20June%202019_0.pdf>. Accessed 2019.
105. World Health Organization Uganda Country Office, Uganda Ministry of Health. In: Ebola Virus Disease Outbreak Uganda Situation Reports. Ebola Virus Disease in Uganda Situation Report 19 June 2019 as of 20 00 Hrs SitRep #08. 2019. <https://www.afro.who.int/sites/default/files/2019-07/Ebola%20Virus%20Disease%20Sitrep%208%2020th%20June%202019_0.pdf>. Accessed 2019.
106. World Health Organization Uganda Country Office, Uganda Ministry of Health. In: Ebola Virus Disease Outbreak Uganda Situation Reports. Ebola Virus Disease in Uganda Situation Report 21 June 2019 as of 20 00 Hrs SitRep #10. 2019. <https://www.afro.who.int/sites/default/files/2019-07/Ebola%20Virus%20Disease%20Sitrep%2010%2021st%20June%202019_0.pdf>. Accessed 2019.
107. World Health Organization Uganda Country Office, Uganda Ministry of Health. In: Ebola Virus Disease Outbreak Uganda Situation Reports. Ebola Virus Disease in Uganda Situation Report 22 June 2019 as of 20 00 Hrs SitRep #11. 2019. <https://www.afro.who.int/sites/default/files/2019-07/Ebola%20Virus%20Disease%20Sitrep%2011%2022nd%20June%202019_0.pdf>. Accessed 2019.
108. World Health Organization Uganda Country Office, Uganda Ministry of Health. In: Ebola Virus Disease Outbreak Uganda Situation Reports. Ebola Virus Disease in Uganda Situation Report 23 June 2019 as of 20 00 Hrs SitRep #12. 2019. <https://www.afro.who.int/sites/default/files/2019-07/Ebola%20Virus%20Disease%20Sitrep%2012%2023rd%20June%202019_0.pdf>. Accessed 2019.
109. World Health Organization Uganda Country Office, Uganda Ministry of Health. In: Ebola Virus Disease Outbreak Uganda Situation Reports. Ebola Virus Disease in Uganda Situation Report 24 June 2019 as of 20 00 Hrs SitRep #13. 2019. <https://www.afro.who.int/sites/default/files/2019-07/Ebola%20Virus%20Disease%20Sitrep%2013%2025th%20June%202019_0.pdf>. Accessed 2019.
110. World Health Organization Uganda Country Office, Uganda Ministry of Health. In: Ebola Virus Disease Outbreak Uganda Situation Reports. Ebola Virus Disease in Uganda Situation Report 25 June 2019 as of 20 00 Hrs SitRep #14. 2019. <https://www.afro.who.int/sites/default/files/2019-07/Ebola%20Virus%20Disease%20Sitrep%2014%2026th%20June%202019_0.pdf>. Accessed 2019.
111. World Health Organization Uganda Country Office, Uganda Ministry of Health. In: Ebola Virus Disease Outbreak Uganda Situation Reports. Ebola Virus Disease in Uganda Situation Report 26 June 2019 as of 20 00 Hrs SitRep #15. 2019. <https://www.afro.who.int/sites/default/files/2019-07/Ebola%20Virus%20Disease%20Sitrep%2015%2027th%20June%202019_0.pdf>. Accessed 2019.
112. World Health Organization Uganda Country Office, Uganda Ministry of Health. In: Ebola Virus Disease Outbreak Uganda Situation Reports. Ebola Virus Disease in Uganda Situation Report 27 June 2019 as of 20 00 Hrs SitRep #16. 2019. <https://www.afro.who.int/sites/default/files/2019-07/Ebola%20Virus%20Disease%20Sitrep%2016%2028th%20June%202019.pdf>. Accessed 2019.
113. World Health Organization Uganda Country Office, Uganda Ministry of Health. In: Ebola Virus Disease Outbreak Uganda Situation Reports. Ebola Virus Disease in Uganda Situation Report 28 June 2019 as of 20 00 Hrs SitRep #17. 2019. <https://www.afro.who.int/sites/default/files/2019-07/Ebola%20Virus%20Disease%20Sitrep%2017_1.pdf>. Accessed 2019.
114. World Health Organization Uganda Country Office, Uganda Ministry of Health. In: Ebola Virus Disease Outbreak Uganda Situation Reports. Ebola Virus Disease in Uganda Situation Report 29 June 2019 as of 20 00 Hrs SitRep #18. 2019. <https://www.afro.who.int/sites/default/files/2019-07/Ebola%20Virus%20Disease%20Sitrep%2018%2029th%20June%202019_1.pdf>. Accessed 2019.
115. World Health Organization Uganda Country Office, Uganda Ministry of Health. In: Ebola Virus Disease Outbreak Uganda Situation Reports. Ebola Virus Disease in Uganda Situation Report 30 June 2019 as of 20:00 Hrs Rep #19. 2019. <https://www.afro.who.int/sites/default/files/2019-07/Ebola%20Virus%20Disease%20Sitrep%2019_1.pdf>. Accessed 2019.
116. World Health Organization Uganda Country Office, Uganda Ministry of Health. In: Ebola Virus Disease Outbreak Uganda Situation Reports. Ebola Virus Disease in Uganda Situation Report 1 July 2019 as of 20:00 Hrs SitRep #20. 2019. <https://www.afro.who.int/sites/default/files/2019-07/Ebola%20Virus%20Disease%20Sitrep%2020_1.pdf>. Accessed 2019.
117. World Health Organization Uganda Country Office, Uganda Ministry of Health. In: Ebola Virus Disease Outbreak Uganda Situation Reports. Ebola Virus Disease in Uganda Situation Report 2 July 2019 as of 20:00 hrs SitRep #21. 2019. <https://www.afro.who.int/sites/default/files/2019-07/Ebola%20Virus%20Disease%20Sitrep%2021%202nd%20July%202019_1.pdf>. Accessed 2019.
118. World Health Organization Uganda Country Office, Uganda Ministry of Health. In: Ebola Virus Disease Outbreak Uganda Situation Reports. Ebola Virus Disease in Uganda Situation Report 3 July 2019 as of 20:00 hrs SitRep #22. 2019. <https://www.afro.who.int/sites/default/files/2019-07/Ebola%20Virus%20Disease%20Sitrep%2022%203rd%20July%202019_1.pdf>. Accessed 2019.
119. World Health Organization Uganda Country Office, Uganda Ministry of Health. In: Ebola Virus Disease Outbreak Uganda Situation Reports. Ebola Virus Disease in Uganda Situation Report 4 July 2019 as of 20:00 hrs SitRep #23. 2019. <https://www.afro.who.int/sites/default/files/2019-07/Ebola%20Virus%20Disease%20Sitrep%2023%204th%20July%202019_1.pdf>. Accessed 2019.
120. World Health Organization Uganda Country Office, Uganda Ministry of Health. In: Ebola Virus Disease Outbreak Uganda Situation Reports. Ebola Virus Disease in Uganda Situation Report 5 July 2019 as of 20:00 hrs SitRep #24. 2019. <https://www.afro.who.int/sites/default/files/2019-07/Ebola%20Virus%20Disease%20Sitrep%2024%205th%20July%202019_1.pdf>. Accessed 2019.
121. World Health Organization Uganda Country Office, Uganda Ministry of Health. In: Ebola Virus Disease Outbreak Uganda Situation Reports. Ebola Virus Disease in Uganda Situation Report 6 July 2019 as of 20:00 hrs SitRep #25. 2019. <https://www.afro.who.int/sites/default/files/2019-07/Ebola%20Virus%20Disease%20Sitrep%2025%206th%20July%202019_1.pdf>. Accessed 2019.
122. World Health Organization Uganda Country Office, Uganda Ministry of Health. In: Ebola Virus Disease Outbreak Uganda Situation Reports. Ebola Virus Disease in Uganda Situation Report 7 July 2019 as of 20:00 hrs SitRep #26. 2019. <https://www.afro.who.int/sites/default/files/2019-07/Ebola%20Virus%20Disease%20Sitrep%2026%207th%20July%202019_1.pdf>. Accessed 2019.
123. World Health Organization Uganda Country Office, Uganda Ministry of Health. In: Ebola Virus Disease Outbreak Uganda Situation Reports. Ebola Virus Disease in Uganda Situation Report 8 July 2019 as of 20:00 hrs SitRep #27. 2019. <https://www.afro.who.int/sites/default/files/2019-07/Ebola%20Virus%20Disease%20Sitrep%2027%208th%20July%202019_1.pdf>. Accessed 2019.
124. World Health Organization Uganda Country Office, Uganda Ministry of Health. In: Ebola Virus Disease Outbreak Uganda Situation Reports. Ebola Virus Disease in Uganda Situation Report 9 July 2019 as of 20:00 hrs SitRep #28. 2019. <https://www.afro.who.int/sites/default/files/2019-07/Ebola%20Virus%20Disease%20Sitrep%2028%209th%20July%202019_1.pdf>. Accessed 2019.
125. World Health Organization Uganda Country Office, Uganda Ministry of Health. In: Ebola Virus Disease Outbreak Uganda Situation Reports. Ebola Virus Disease in Uganda Situation Report 10 July 2019 as of 20:00 hrs SitRep #29. 2019. <https://www.afro.who.int/sites/default/files/2019-07/Ebola%20Virus%20Disease%20Sitrep%2029%2010th%20July%202019_0.pdf>. Accessed 2019.
126. World Health Organization Uganda Country Office, Uganda Ministry of Health. In: Ebola Virus Disease Outbreak Uganda Situation Reports. Ebola Virus Disease in Uganda Situation Report 11 July 2019 as of 20:00 hrs SitRep #30. 2019. <https://www.afro.who.int/sites/default/files/2019-07/Ebola%20Virus%20Disease%20Sitrep%2030%2011th%20July%202019_1.pdf>. Accessed 2019.
127. World Health Organization Uganda Country Office, Uganda Ministry of Health. In: Ebola Virus Disease Outbreak Uganda Situation Reports. Ebola Virus Disease in Uganda Situation Report 12 July 2019 as of 20:00 hrs SitRep #31. 2019. <https://www.afro.who.int/sites/default/files/2019-07/EVD_Kasese_Sitrep_%2331_1.pdf>. Accessed 2019.
128. World Health Organization Uganda Country Office, Uganda Ministry of Health. In: Ebola Virus Disease Outbreak Uganda Situation Reports. Ebola Virus Disease in Uganda Situation Report 14 July 2019 as of 20:00 hrs SitRep #33. 2019. <https://www.afro.who.int/sites/default/files/2019-07/EVD_Kasese_Sitrep_%2333_1.pdf>. Accessed 2019.
129. World Health Organization Uganda Country Office, Uganda Ministry of Health. In: Ebola Virus Disease Outbreak Uganda Situation Reports. Ebola Virus Disease in Uganda Situation Report 15 July 2019 as of 20 00 Hrs SitRep #34. 2019. <https://www.afro.who.int/sites/default/files/2019-07/EVD_Kasese_Sitrep_%2334_1.pdf>. Accessed 2019.
130. World Health Organization Uganda Country Office, Uganda Ministry of Health. Joint Press Release: Joint Advisory on Ebola Virus Disease in Uganda. 2019. <https://www.health.go.ug/download/file/fid/2331>. Accessed 2019.
131. World Health Organization Uganda Country Office, Uganda Ministry of Health. Joint Press Release: Clarification on the movements of an Ebola Virus Disease case who recently died in Democratic Republic of Congo (DRC). 2019. <https://www.afro.who.int/sites/default/files/2019-07/Joint%20Release%20-%20Clarification%20on%20the%20movements%20of%20an%20EVD%20case%20who%20died%20in%20DRC.PDF>. Accessed 2019.
132. Uganda Office of the Minister of State of Health – Primary Health Care – Public Relations Unit. Press Release: Confirmation of an Imported Ebola Virus Disease Case in Kasese District. 2019. <https://www.health.go.ug/document/confirmation-of-an-imported-ebola-virus-disease-case-in-kasese-district/>. Accessed 2019.
133. Uganda Office of the Minister of State of Health – Primary Health Care – Public Relations Unit. Press Release: Update of Ebola Outbreak in Kasese District. 2019. <https://reliefweb.int/report/uganda/update-ebola-outbreak-kasese-district-21-june-2019>. Accessed 2019.
134. World Health Organization Uganda Country Office, Uganda Ministry of Health. Joint Press Release: Uganda declares end of Ebola outbreak. 2019. <https://www.health.go.ug/download/file/fid/2462>. Accessed 2019.
135. World Health Organization Uganda Country Office, Uganda Ministry of Health. Joint Press Release: Weekly Travel Advisory on Ebola Virus Disease in Uganda. 2019. <https://www.health.go.ug/document/weekly-travel-advisory-on-ebola-virus-disease-in-uganda/>. Accessed 2019.
136. Uganda Office of Director General – Public Relations Unit. Press Release: No Ebola in Mbarara District. 2019. <https://www.health.go.ug/download/file/fid/2305>. Accessed 2019.
137. Uganda Ministry of Health. Press statement on Ebola outbreak in Kasese District. 2019. <https://www.health.go.ug/download/file/fid/2276>. Accessed 2019.
138. Uganda Ministry of Health. Press statement on the Imported Ebola Virus Disease (EVD) Case in Kasese District. 2019. <https://www.health.go.ug/document/press-statement-on-the-imported-ebola-virus-disease-evd-case-in-kasese-district/>. Accessed 2019.
139. Uganda Office of the Ministry of Health: Press release: Update of Ebola Outbreak in Kasese District. 2019. <https://reliefweb.int/report/uganda/update-ebola-outbreak-kasese-district-13-june-2019>. Accessed 2019.
140. World Health Organization Uganda Country Office, Uganda Ministry of Health. Joint Press Release: WHO Director General Visits Uganda to Assess Ebola Response. 2019. <https://www.afro.who.int/news/who-director-general-visits-uganda-assess-ebola-response>. Accessed 2019.
141. World Health Organization Uganda Country Office, Uganda Ministry of Health. Joint Press Release: Update on DRC Trader Who Visited Uganda, Later Died of Ebola in DRC. 2019. <https://www.health.go.ug/document/update-on-drc-trader-who-visited-uganda-later-died-of-ebola-in-drc/>. Accessed 2019.
142. World Health Organization Uganda Country Office, Uganda Ministry of Health. Joint Press Release: Weekly Travel Advisory on Ebola Virus Disease in Uganda. 2019. <https://www.health.go.ug/document/weekly-travel-advisory-on-ebola-virus-disease-in-uganda-2/>. Accessed 2019.
143. World Health Organization. Confirmation of case of ebola virus disease in Uganda. 2019. <https://www.afro.who.int/news/confirmation-case-ebola-virus-disease-uganda>. Accessed 2019.
