# Supplementary material for: Retrospective identification of key activities in Uganda’s preparedness measures related to the 2018–2020 EVD outbreak in eastern DRC utilizing a framework evaluation tool
Source: PLOS Glob Public Health. 2022 May 11;2(5):e0000428. doi: 10.1371/journal.pgph.0000428 (PMC10021806; doi:10.1371/journal.pgph.0000428)
Supplement: S2 Text — Please, see semi-structured interview guide utilized for key informant interviews. (DOCX) [file pgph.0000428.s002.docx]

**INTERVIEW GUIDE**

This interview guide is meant to help facilitate discussion between the researcher and the interviewee, but is does not need to be strictly adhered to or systematically followed during each interview. Each interviewee will provide a unique perspective on Uganda’s Ebola outbreak preparedness efforts. Individual interviewees may or may not have the ability to address all of these questions, and they will likely identify additional topics or themes not included in this guide that they feel are important aspects of their experience.

***Project description*** (to be provided to the interviewee prior to the start of the interview): This project is a collaboration between Makerere University and the Johns Hopkins Center for Health Security’s Outbreak Observatory. We are interested in collecting the challenges and lessons from Uganda’s efforts to increase capacity to prevent and respond to an Ebola outbreak with the ultimate goal of helping other countries who endeavor to prepare for similar infectious disease outbreaks. We are interviewing individuals involved in various efforts to prevent the importation of Ebola into Uganda from the Democratic Republic of Congo (DRC), including officials from the Ministry of Health, public health practitioners, community leaders, and local healthcare workers. With your permission, notes will be taken during the interview to ensure accuracy when reporting findings. Once the interviews are complete, the research team will analyze the topics and details discussed in the interviews to identify and characterize key themes and compose a manuscript that will be submitted to a peer-reviewed journal.

***General interview questions***

- What has been your role during the response to the ongoing outbreak of Ebola in DRC?
- Can you describe some of the strategies, activities, policies, and/or programs that Uganda has implemented to prevent the spread of Ebola across the DRC border?
  - Which of these do you feel are the most successful or impactful? Why?
  - Can you discuss any challenges faced during Uganda’s Ebola preparedness efforts?
    - How has Uganda adapted preparedness strategies in response to these challenges
  - What resources have these activities required?
  - Are there any outstanding gaps in Uganda’s Ebola preparedness?
- What surveillance strategies are you using to identify potential Ebola cases?
- We understand that the Uganda Ministry of Health recently conducted a simulation exercise for Ebola virus prevention and control. Did you participate in this exercise?
  - Do you believe this simulation was helpful?
  - What did you learn?
  - How can these lessons learned be integrated into the current and future responses?
- How has the current response differed from Uganda’s response to the 2013-16 Ebola epidemic in West Africa?
  - Were there specific lessons learned during the 2014 response that have been integrated into the current response?
- Has the need to prepare for a potential Ebola outbreak impacted other preparedness efforts?
  - For example, has Ebola preparedness drawn resources away from other programs, such as the Joint External Evaluation process, or has it hindered your ability to address gaps highlighted in your National Action Plan for Health Security?

***For those involved in border screening***

- Can you please explain Uganda’s process for border screening and/or the process that follows for those who are identified as being at risk for Ebola?
  - What types of screening questions/criteria are being used (eg, travel history, symptoms)?
  - To your knowledge, when did Uganda begin conducting border screenings?
  - To your knowledge, is Uganda continuing to monitor people once they cross the border?
  - Where is Uganda conducting border screening? Are there areas of concern that are currently not covered by border screening (ie, can individuals avoid specific checkpoints and cross the border without being screened)?
- What are some of the challenges and success that you have learned from conducting border screenings that might be useful for other countries to know during future responses?
  - For example, how have you addressed the challenge of individuals who may cross the border without passing through immigration controls?
  - How have these challenges resulted in changes to Uganda’s border screening process/program?
- Do you believe that border screenings have been useful in preventing the spread of Ebola across the border from DRC into Uganda?
- What resources (eg, number of personnel, supplies, financial costs) are required to conduct border screening?
- Do you know how many people have been screened up until the time of this interview?
  - How many have been identified as at risk for exposure or as a person under investigation (PUI)?
- Are there any other lessons or gaps in Uganda’s border screening that you would like to discuss?

***For those involved in vaccination healthcare workers (HCWs)***

- How is Uganda identifying priority healthcare workers for vaccination?
  - Which categories of healthcare workers are being prioritized (eg, those closest to the border, those who work in a specific specialty, infection control, environmental cleaning/sanitation)?
  - When did you begin to vaccinate healthcare workers?
    - How many healthcare workers have been vaccinated?
    - How many more vaccinations does Uganda anticipate administering?
- What have been some of the challenges of vaccinating healthcare workers, and how have you addressed them?
  - In particular, have there been any challenges in the context of participating in ongoing clinical trials for the investigational vaccines?
- Do you believe that vaccination of healthcare workers has been useful in preventing the spread of Ebola across the border from DRC into Uganda?
- What resources have been required to vaccinate healthcare workers?
- How has Uganda procured the vaccines to support healthcare worker vaccination?
  - Has Uganda utilized existing vaccine distribution plans/networks, or were plans developed specifically for the Ebola vaccines?

***For those involved in the healthcare sector***

- How have you helped to prepare healthcare workers for Ebola cases (eg, training on personal protective equipment, training on reporting policies, safe burials, psychosocial support)?
  - Are you confident that healthcare workers will be able to recognize an Ebola case?
- How have you increased capacity in the health sector to respond to an Ebola outbreak (eg, vaccination teams, building isolation units, procuring supplies)?
  - How did you identify these efforts as the priority activities to increase response capacity for Uganda?
  - What resources have been required to improve preparedness and response capacity?
  - Are there plans to sustain the increased capacity for future use, or are these efforts in place until the threat of Ebola crossing the border is no longer apparent?

***For those involved in community outreach and engagement***

- Has Uganda prioritized specific communities or subpopulations for engagement? If so, which ones?
- How has Uganda identified community leaders and other influential individuals(eg, teachers, religious leaders, healers, local government leaders)?
- How were educational messages developed and disseminated (eg, television and radio, posters, pamphlets)?
- Do you believe engaging with the community and providing educational messages has increased Uganda’s Ebola preparedness?
- What other activities have you engaged in with communities to prevent the spread of Ebola (eg, orientation packages for teachers on Ebola prevention, detection and control measures)?
- What resources were required for community engagement?

***For those involved in public communication***

- Please describe the efforts Uganda has taken to communicate directly with the public about Ebola and the risk of importation from DRC.
  - What methods have you used to communicate with the public?
  - What have your key messages been to the public? Have they focused on reporting? Not traveling? Monitoring symptoms?
  - Are there specific approaches that Uganda has taken that could be helpful for other countries to know when responding to an Ebola outbreak?
- What have some of the challenges been in communicating with the public, and how have you addressed them?
- Did you face any hostility, opposition, or noncooperation for the general public or specific communities? If so, how did you address this?
- Were there any other challenges or lessons in the context of public communication or community outreach activities? Where there any changes to Uganda’s communication efforts or community outreach processes in light of these challenges?
